# Typhlitis

**Published:** 1889-01

**Authors:** Thos. D. Wooten

**Affiliations:** Austin, Texas


DANIEL'S

### TEXAS MEDICAL JOURNAL.

A Representative Organ of the Medical Profession, and an Exponent of Rational Medicine; devoted to the Organization, Advancement and Elevation of the Profession in Texas.

PUBLISHED MONTHLY.—SUBSCRIPTION $2.00 A YEAR.

Vol. IV. AUSTIN, JANUARY, 1889. No. 7.

### ORIGINAL ARTICLES.

CONTRIBUTED EXCLUSIVELY TO THIS JOURNAL.

*The Articles in this Department are accepted and published with the understanding that we are not responsible for, nor do we indorse the views and opinions of the writers by so doing.*

### TYPHLITIS.

BY THOS. D. WOOTEN, M. D., AUSTIN, TEXAS.

[Read before the Austin District Medical Society, December 20, 1888, and voted to DANIEL'S TEXAS MEDICAL JOURNAL for publication.]

FROM allusions made by some of the oldest medical writers, we are led to infer that this disease was observed at an early period in the history of medicine, though without the knowledge of the nature of the processes involved. Sautorini describes fecal accumulations affecting the caecum in 1715. P. Frank describes perityphlitis as peritonitis muscularis. Dupuytren seems to have been first to individualize this disease as a distinct affection. Stokes, in 1837, wrote upon this disease, calling attention to the value of opium in perforations of the appendix, since which time numerous observers have written upon the disease, until the present literature of the subject is quite extensive, furnishing a broad field of observation and experience, a thorough study and knowledge of which, by a student, leaves but little wanting to acquaint him with its nature, causation, progress and treatment.

This paper, however, proposes to give only a slight synopsis of a practical character relating to the disease, culled from the literature accessible to its author, with the view of bringing the subject before the Society for discussion, rather than an extended disquisition on the subject; particularly since my own personal observation and experience have been limited, I can add nothing of value from this source.

The caecum is the large blind pouch, or cul-de-sac in which the colon commences; it is the most dilated part of the colon, measuring from two to two and a half inches vertically and transversely. It is situated in the right iliac fossa, to which it is attached by loose areola tissue. Occasionally it is almost surrounded by peritoneum, forming a distinct fold, which condition affords considerable freedom of movement.

Attached to its lower and back part is the appendix vermiformis, a long, narrow, worm-shaped tube, in length from three to six inches, in diameter, the size of a goose quill. It is usually directed upward and downward behind the caecum, coiled upon itself, and terminates in a blunt point. It communicates with the caecum by an orifice which is sometimes guarded by an incomplete valve.

The function of the appendix vermiformis is a matter of conjecture. It is usually found filled with a vitrious mucus throughout its length. The existence of this superfluous structure, which is only found in man and the higher order of apes, has given rise to much conjecture and speculation. It is an acknowledged opinion of many physiologists of the texlogical school that the appendix vermiformis is a relic or rudiment of a subsidiary stomach in the lower forms of animal life. The caecum forms almost a second stomach in the gramnivora, and is three times the length of the whole body in the marsupial Kaola, and is found much smaller in the carnivora, whose food contains but little indigestible matter.

In the omnivora, as we find it in man, the vermiform appendix is the shriveled remains of the great cœcal receptaculum of of lower animals. In the primeval state of man, when fed as did Nebuchadnezzar—in his latter days, it may have been an important factor in his digestive process; with man's present cuisine and civilized mode of life, the vermiform process is characterized by pathologists with the significant appellation of "death trap."

To make out clearly a case of typhlitis it is important to be able to localize and determine the anatomical relations of the organ involved. According to Laschka, "the caecum lies in such a manner on the right iliac muscle that its extremity corresponds with the middle of Poupart's ligament, it lies just above the external half of this latter, on the inner portion of the anterior abdominal wall. In exceptional cases the caecum assumes a higher position or is turned around toward the entrance of the true pelvis, and may even extend across the middle line. The caecum is not in immediate contact with the iliacus muscle, as it possesses a complete peritoneal investment, so that when distended it projects freely into the peritoneal cavity. This portion of intestine presents a wedged shaped form, with its end pointed or truncated, according as the vermiform process opens into the true end, or enters in such a way that the connections to all appearances are lost. The relation of the caecum to the iliacus muscle is such that every time the thigh is bent it presses up and stimulates this portion of the intestine."

Typhlitis, strictly speaking, is limited to affections of the caecum and its appendix vermiformis. Perityphlitis is mostly due to extension of the inflammation to the peritoneal envelope of these organs, while paratyphlitis signifies an involvement of the extraperitoneal and post-cœcal connection tissues. Both paratyphlitis and perityphlitis are therefore (ordinarily) secondary processes. (Pepper.)

According to all statisticians it is a disease having an especial prediliction for the male sex, and the period of adolescence.

Nearly three-fourths of the whole number of cases tabulated have so occurred. The greatest number of cases, 33 per cent., have occurred between the ages of 21 and 30; next, 30 per cent., at 11 to 20, while the ratio decreases toward both extremes of life. Typhlitis seems so often to arise from disease of the appendix, it calls for especial attention in the etiology of the disease. Five per cent. of all post mortem cases, we are told, show evidences of it having been deseased; especially does it occur in subjects of tuberculosis, dysentery and typhoid fever.

The valvular fold of the mucous membrane at the orifice of the tube, and the absence of muscular fibers in the walls of the process rendering it incapable of emptying its contents, and being always filled with mucus, and together with the stagnant condition of the adjacent caecum, they afford a most eligible nidus, or site, for the lodgment of foreign bodies, germs, or virus of disease taken with the food or drink, and traversing inocuously the whole alimentary tract to bring up at this quiet habitat for injury or development or rapid reproduction. Additional factors doubtless arise from congenital defects, abnormal anatomical position and former disease. Post mortems have shown abnormalities to be quite frequent. Constipation is regarded by all authority as a prominent factor in the production of typhlitis, whether it be due to the character of food or want of peristalsis or paresis of the muscular coat of the caecum, resulting from intestinal catarrh, of disease virus, fecal concretions or foreign bodies, conditions that may give rise to spasmodic action followed by paresis, or such a condition of partial paresis may arise from inhibition of nerve force reflected from some other point of irritation.

The postmortem table shows the morbid anatomy for the most part, to be the picture of perforative peritonitis, which is the usual cause of death. The peritoneum in the viciuity of the perforation presents the changes incident to inflammation, s uch as hyperaemia, swollen necrosed, flakes of fibrin, partially agglutinated to contiguous structures. The bowel very much thickened, catarrhal swelling of the mucosa, proliferation of its submucous tissues; not unfrequently the muscous tissue is the seat of extensive ulcerations and swolen adjacent structures, or there forms an abscess as large as a man's head, in its immediate vicinity. The abscess may remain strictly localized, or it may wander and discharge into ilium, caecum, duodenum, colon, diaphragm, bladder inferior vena cava, abscess of the lower pleural cavity, and other points, as has been cited by various authorities; into the peritoneal savity most frequently, and with the most serious consequences. In cases running a less active course, the lesions are found about the appendix. Various contortions, adhesions and erosions are centered upon the structure, and may be found perforated in several places, cicatrices agglutinations evidences of former disease; in some instances the organ was partially, and in others entirely destroyed and absorbed. (Bauer.) The symptoms in this disease are variable, determined by the primary seat of the inflammation, the exciting cause and the extent of the pathological changes; conditions which also influence largely the course and progress of the disease. The more rapidly and extensively the peritoneum becomes imvolved, the more rapid and virulent the course of the disease. If the serous membrane is circumscribed in its involement, the disease may be latent and slow in its progress.

The changes that occur in the vermiform process and caecum, which may lead to peritonitis, often remain latent, and manifest their existence only by disturbance of the bowels; constipation may have existed for a long time, or followed by an attack of diarrhœa, digestion disturbed for a period of time attended with pain and tenderness in the region of caecum and ascending colon. This latent manifestation ot symptoms by reason of imprudence, or wanted medical attention, may develop into acute and virulent symptoms; qnite often, however, virulent manifestations of the inflammatory process is developed suddenly in the right iliac fossa, accompanied by severe pain, shivering, followed by fever and subsequently the fever is determined by the inflammation process. The most important physical sign is a tumor or enlargement in the position of caecum, and may extend up the colon. It usually projects out prominently into the peritoneal cavity, and is directly observable through the abdominal wall, a short distance above Poupart's ligament.

The enlargement of the caecum in all cases extends inward beyond its normal limits. The tumor at its inner margin may extend to the middle line. This is determined by its position of projecting into the peritoneal cavity and the resistance offered by the ileum on the outer margin. The tumor feels comparatively superficial. It is only slightly movable or not movable at all. It is usually sharply defined in its marginal circumference. The surface is usually smooth and more or less firm and resisting. Fluctuation is but rarely observable, and then only in those cases where the contents are about to burst outward, The percussion sound is either perfectly dull or dully tympanitic.

My own limited observation leads me both to approve and appreciate the opinion expressed by Bauer, in which he says: "The caecum and vermiform process are often the seat of disease, which leads to circumscribed peritonitis. The connecting tissue also which lies behind the caecum over iliac fossa, is sometimes the seat of inflammation and abscess. The first mentioned processes are from the outset intraperitoneal, while phlegmonous inflammation in the areola tissue behind the .caecum is first retroperitoneal, but frequently involves the peritoneum. and may secondarilly extend to the intestinal wall. These inflammatory processes are therefore anatomically distinct in their position, and they also possess from the outset a different significance and importance, nevertheless, they present in their symptoms so much in uniformity that the clinical distinction between them in the majority of cases is impossible. We must be satisfied with recognizing the inflammation in the right iliac fossa, without distinguishing whether the caecum, the appendix, or the retroperitoneal areola tissue be the starting point; in many cases, furthermore, the distinction is impossible, it being at the same time, however, a matter of no practical importance, owing to the fact that the forms of inflammation mentioned, pass into one another so, that by an ulceration of the vermiform process, the caecum becomes involved." This tumor or enlargement is usually largely due to the accumulation of feces in the caecum, it is indeed a frequent occurrence, due most likely to paralysis of the muscular coat of the bowel from pressure, effusion from without in the vicinity, and the abnormality of position promoted by the primary disease of the caecum or appendix. This impaction or accumulation of feces may result in complete occlusion of the bowel leading to ilius above, while diarrhœa from catarrh of the large bowel may exist below, though complete occlusion at the caecum exist. In other cases a partial passage is left by the side of the fecal mass. Stercoracious vomiting is usual in such conditions of occlusion; gastric vomiting is a common symptom in all forms of the disease. That impacted feces may exist as a primary cause and condition giving rise to typhlitis, is evident from authorities, and is styled typhlitis stercoralis. It is indicated by a well defined tumor from the commencement, and may arise from the character of food and habits of the individual; an enlargement from impaction may attain a large size, but is wanting in the sensitiveness and pain incident to that dependant upon primary inflammation of caecum. In the latter cases there is merely fullness in the early stage and afterwards a distinct tumor. When the tumor arises from inflammatory thickening of the caecum, it is less dull on percussion, than if due to fæcal accumulation, and a complete timely removal of the impaction usually terminates the trouble. An attack of typhlitis may last from two or three to twelve days and subside by resolution, the bowels are copiously relieved, the vomiting ceasing, the pain tenderness, and tumor disappearing from the right iliac fossa. The gravity of the symptoms after creating much alarm and anxiety may thus terminate favorably. Often, however, the termination is not so satisfactory, the course becomes tedious and dangerous. The inflammation becoming general by its extension, or an abscess forms and breaks into the peritoneal cavity, or makes its escape elsewhere; recovery under such circumstances, if at all, must be after a tedious and protracted illness covering many weeks if not months. Typhlitis terminates much more rapidly in children; they succumb to the disease often in a very few days. According to Matterstock, 44 per cent. of children die from the disease within the first three days. The average duration of the disease without suppuration, is from 14 to 20 days. Prognosis: Pepper says typhlitis stercoralis has no gravity and should terminate, or be terminated within twenty-four or twenty-eight hours after its recognition. Neglected or unrecognized cases are not unfrequently fatal. Typhlitis independent of fecal impaction is always a grave affection. The mortality alone in childhood is 70 per cent, in adult 30 per cent. Since the adoption of the opium treatment the mortality has been reduced from 80 to 30 per cent. in adults.

The treatment of typhlitis should receive earnest consideration and careful discrimination as to the cause and forms of the disease, as the several pathological conditions suggest very different therapeutic indications. Typhlitis stercoralis, for instance, calls for evacuation of the impacted feces; while peri or paratyphlitis suggest a line of treatment giving functional rest and quiet to the bowels, such as we recognize as indicated in impending peritonitis. To fulfil the indications in typhlitis stercoralis, the simplest, most effective and probably the most potent means at our command of dislodging the impacted feces, is by irrigation properly and efficiently employed; this may be accomplished by the agency of a fountain syringe or rubber tubing several feet in length attached to a funnel or an irrigating bottle, the other end having a proper attachment to enter the rectum, which may consist of a large elastic male catheter or tube of sufficient elasticity and strength to be carried up above the sigmoid flexure, or one such as is used upon an ordinary anal syringe; the patient being placed upon the right side with hips much elevated, or in the knee and chest position, the latter preferable; in such position, the patient if weak and feeble, should be well and comfortably supported; then by a slow, and as much as possible by a painless process, fill the entire length of the colon with warm soft water, which may contain glycerine, or a limited quantity of sulphate magnesia or both; no undue force should be used by over elevation of the tube, but rather accomplish the end sought by the gentle force of percolation and decent of the fluid; a few well directed procedures of this kind will accomplish the dislodgement of the fecal impaction of the intestine. Auxiliary to, and during the intervals of irrigation, half a drachm or a drachm of sulph. magnesia may be administered in some agreeable menstruum every hour or two, not so much with the view of exciting peristalsis, as producing exosmosis and liquifying the contents of the bowels above.

As before suggested, the other forms of the disease suggest remedial agencies that will put at rest the irregular, spasmodic and tetanic contractions of the muscular coat of the bowel and the reflexed action upon the muscles of the abdominal walls. The physiological action of opium has been found to fulfil more efficiently this indication than any other known agent. It should be given in such quantity and frequency as to accomplish the purpose in view, yet with such a degree of caution, especially with children, as not to produce its toxic effect, which is liable to supervene upon the sudden cessation of pain. I believe it is conceded that opium in its crude form, or in liquid form is more effective in this disease, as it is found to be in peritonitis. The remedial action of opium in typhlitis is due to its effects in allaying and quieting mnscular action, thereby favoring adhesions and the consequent limitation of peritonitis, and the products of the inflammatory process, and suggest in addition complete rest of the body. The ice bag has been found effective in the inccption of the disease, attended with fever and a sthenic condition.

The ice bag should be shifted in its position or suspended, to avoid undue contact and pressure. In a more advanced stage of the disease warm fomentations and poultices applied over a large extent of abdominal surface will be found more agreeable, and afford greater comfort to the patient. Gentle friction with mercurial ointment or iodine have been found beneficial. As soon as systemic and local manifestations of the formation and existence of pus is indicated by a distinct doughey sensation, or a more decided fluctuation, early and prompt means should be used for its evacuation. If doubt should remain of its existence, an aspirating needle of large size may be used to clear np the diagnosis; great relief is often afforded by aspiration when but a small quantity of serous or purulent fluid escapes. Aspiration may be repeated until the pus is evacuated. Should the introduction of the needle disclose the existence of pus, and it is thought best to evacuate by incision and establish drainage, the needle should be retained and used as a guide for the incision. Abscesses superficial and large in size, or of continuous formation should be relieved by free incision, drainage and complete disinfection. An incision, however, should not be made too early, the adhesion may not have occurred, and as a consequence the contents of the abscess or caecum may escape into the peritoneum and produce the very result that early evacuation is intended to prevent. The early removal of the products of inflammation lessens the serious danger of perforative peritonitis, and a consequent fatal termination. It also prevents the formation of burrowing sinuses, fistulous openings leading to amyloid degeneration and marasmus.

In occlusion of the bowel irrigation of the stomach has recently been claimed as beneficial, as stated by Bartholomew. I can hardly understand its mode of relief.

The diet, hygienic surroundings, etc. of the patient require no comment, as they are clearly manifest by the nature of the case. In case of perforation opium is indicated to localize peritonitis by encapsulating the matter discharged. An early and prompt laparotomy is justifiable and indicated and should be made available in all cases in which the general condition of the patient gives any warrant or reasonable hope of success.

